# Recognition of out-of-hospital cardiac arrest during emergency calls — a systematic review of observational studies

**DOI:** 10.1186/s13049-017-0350-8

**Published:** 2017-02-01

**Authors:** Søren Viereck, Thea Palsgaard Møller, Josephine Philip Rothman, Fredrik Folke, Freddy Knudsen Lippert

**Affiliations:** 1 Emergency Medical Services Copenhagen, University of Copenhagen, Telegrafvej 5, DK-2750 Copenhagen, Denmark; 2 Center for Perioperative Optimization, Department of Surgery, Herlev Hospital, University of Copenhagen, Herlev Ringvej 75, DK-2730 Copenhagen, Denmark

**Keywords:** Out-of-hospital cardiac arrest, Emergency medical services, Emergency medical dispatch, Systematic review, Uniform reporting, Key performance indicator

## Abstract

**Background:**

The medical dispatcher plays an essential role as part of the first link in the Chain of Survival, by recognising the out-of-hospital cardiac arrest (OHCA) during the emergency call, dispatching the appropriate first responder or emergency medical services response, performing dispatcher assisted cardiopulmonary resuscitation, and referring to the nearest automated external defibrillator. The objective of this systematic review was to evaluate and compare studies reporting recognition of OHCA patients during emergency calls.

**Methods:**

This systematic review was reported in compliance with the PRISMA guidelines. We systematically searched MEDLINE, Embase and the Cochrane Library on 4 November 2015. Observational studies, reporting the proportion of clinically confirmed OHCAs that was recognised during the emergency call, were included. Two authors independently screened abstracts and full-text articles for inclusion. Data were extracted and the risk of bias within studies was assessed using the QUADAS-2 tool for quality assessment of diagnostic accuracy studies.

**Results:**

A total of 3,180 abstracts were screened for eligibility and 53 publications were assessed in full-text. We identified 16 studies including 6,955 patients that fulfilled the criteria for inclusion in the systematic review. The studies reported recognition of OHCA with a median sensitivity of 73.9% (range: 14.1–96.9%). The selection of study population and the definition of “recognised OHCA” (threshold for positive test) varied greatly between the studies, resulting in high risk of bias. Heterogeneity in the studies precluded meta-analysis.

**Conclusion:**

Among the 16 included studies, we found a median sensitivity for OHCA recognition of 73.9% (range: 14.1–96.9%). However, great heterogeneity between study populations and in the definition of “recognised OHCA”, lead to insufficient comparability of results. Uniform and transparent reporting is required to ensure comparability and development towards best practice.

## Background

Rapid initiation of bystander cardiopulmonary resuscitation (CPR) and early defibrillation are significant predictors for survival of out-of-hospital cardiac arrest (OHCA). [1–3] Especially in Denmark and Sweden where we have seen substantial increases in bystander CPR resulting in improved survival. [1, 3] Success in the first link in the chain of survival – the recognition of OHCA by bystanders or medical dispatchers – is crucial for the activation of the subsequent links.[4, 5] Recognition of OHCA during the emergency call is a prerequisite for the provision of dispatcher-assisted CPR instructions and can also increase the activation of public access defibrillation programmes – initiatives that can improve the chance of bystander CPR and long-term survival.[6–12] Despite several initiatives to improve bystander CPR and automated external defibrillator (AED) use, survival remains low.[2, 13] Uniform reporting of measurements in medical dispatch may hold the potential to improve the the performance in the first link of the Chain of Survival.

As part of improving the first link in the chain of survival and ultimately increase survival, recognition of OHCA could be used as a key performance indicator for comparison of EMS systems. However, few studies have reported the recognition of OHCA and results have been of great variation.[14, 15] The reason for this may be variety in competences across EMS systems or unequal registration of OHCA recognition. The importance of recognition of OHCA during the emergency call, has been emphasised in the European Resuscitation Council 2015 guidelines, however, no framework for reporting recognition has been implemented. To use recognition of OHCA as a key performance indicator for comparison of EMS systems and thereby gain knowledge across systems, it is critical that results are reported in a uniform and transparent way.

The objective of this systematic review was to evaluate and compare studies reporting recognition of OHCA patients during emergency calls.

## Methods

This systematic review was conducted and reported according to the PRISMA-guidelines and the protocol was registered in the PROSPERO database (CRD42014010638).[16].

### Eligibility criteria

The research question for the literature search in this systematic review was created using the PICOS model as recommended in the Cochrane Handbook[17]: P = Population: Patients suffering from OHCA of any origin; I = Intervention: Non applicable as no interventions were studied; C = Comparison: Non applicable as no interventions were studied; O = Outcome: Proportion of OHCAs recognised during the emergency call (recognition); S = Studies: Observational studies. We included published English-language observational studies with no publication date restrictions. Studies were considered eligible for analysis, if the sensitivity of OHCA recognition, during emergency calls of clinically confirmed OHCA, was calculated and reported. Observational studies reporting OHCA recognition before and after an intervention were excluded. Case reports, conference abstracts, letters, and published protocols were also excluded.

Several studies defined “recognition of OHCA” as the amount of cases dispatched as OHCA that had a final diagnosis of OHCA. They essentially report the positive predictive value (PPV) of OHCA recognition and not the sensitivity. It is not possible to calculate the sensitivity of OHCA recognition in these studies, as they did not report data on the false negatives cases. With no reported sensitivity, or the possibility to calculate sensitivity, these studies did not match the pre-specified inclusion criteria for this systematic review, and were therefore excluded.

### Information sources

**Fig. 1:**
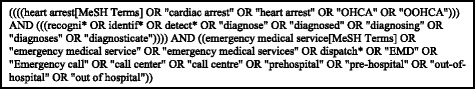
MEDLINE search strategy

We systematically searched MEDLINE, Embase, and The Cochrane Library on 4 November 2015. The search strategy was planned in collaboration with a librarian. The search-strategy from MEDLINE (Fig. 1) was modified to fit Embase and The Cochrane Library.

### Study selection

We removed duplicates occurring in more than one database. Studies were screened by title and abstract by two authors (SV and JPR) independently. Interrater reliability for the screening of abstracts was calculated using Cohen’s Kappa statistics. Included records were assessed for eligibility in full-text by two authors (SV and TPM) independently. Any discrepancies were solved by discussion until consensus. Finally, reference lists of included full-text studies were screened for studies that fulfilled the inclusion criteria.

### Data collection process

The included studies were analysed and data were extracted into separate, predetermined tables by the first author. The collected data were:

- Study setting: Study period, country/state, study design, inhabitants, data sources
- EMS characteristics: Decision tool, dispatcher-assisted CPR instructions, education/qualification of emergency medical dispatchers
- Methodology: Definition of study population, definition of "recognised cardiac arrest”, type of registration for recognition
- Quantitative measures for recognition: Number of clinically OHCA analysed, false positive, incidence of OHCA analysed/100,000/year, sensitivity, positive predictive value

### Risk of bias in individual studies

Since the recognition of OHCA during emergency calls can be evaluated as a diagnostic test, we assessed risk of bias using the QUADAS-2 tool for quality assessment of diagnostic accuracy studies.[18] The QUADAS-2 tool rates the risk of bias as “Low”, “High” or “Unclear” by evaluating four key domains: “Patient Selection”, “Index Test”, “Reference Standard”, and “Flow and Timing”. The QUADAS-2 tool can also be used to assess concerns regarding applicability; however, in this review we assessed applicability in the full text evaluations. The signalling questions in the tool were adjusted to the review, and review-specific guidance on how to assess the signalling questions was developed as recommended. The refined tool was piloted on three random studies by two authors (SV and JPR), and after good agreement, one author (SV) completed the quality assessment for all studies.

### Summary measures

The principal summary measures used for comparison of recognition was sensitivity (the proportion of clinically confirmed OHCAs recognised by the medical dispatcher) and the positive predictive value (the amount of cases dispatched as OHCA, that were confirmed as clinical OHCA; PPV), as well as the incidence of OHCA analysed/100,000/year in the study population. The authors calculated the PPV in studies that only reported the amount of false positives and true positives. One study did not provide precise information about study area or total inhabitants [19], therefore it was not possible to calculate the incidence of OHCA. Another study only provided information about study area.[6] In this case the number of inhabitants was extracted from the United States Census Bureau as a cut-off by the study period, and the incidence of OHCA was calculated.[20].

## Results

**Fig. 2:**
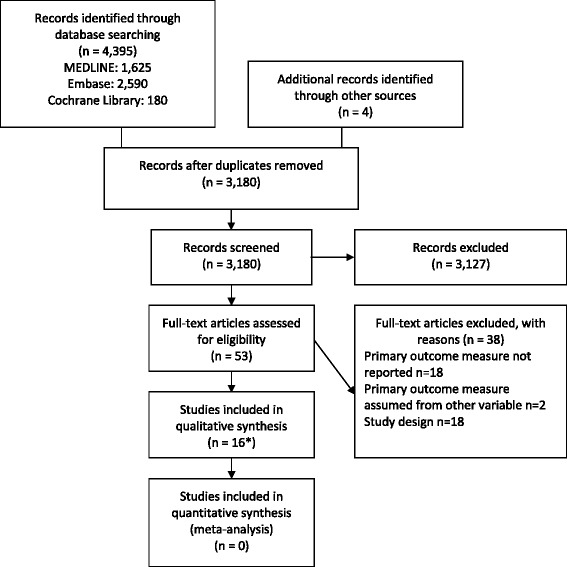
PRISMA flowchart. Flowchart describing the study selection process, *One publication presented results from two different EMS systems in two separate countries. In this case, both sets of results were included, as they were collected and analysed independently

In total, 4,395 studies were identified through database searches. After removal of duplicates, 3,180 abstracts were screened for eligibility and 53 were assessed in full-text. Four additional studies were identified through the search of reference lists of included studies. Ultimately 15 publications, with a total of 6,955 patients, met the inclusion criteria and were included in the systematic review (Fig. 2). One publication presented results from two different EMS systems in two separate countries.[21] In this case, both sets of results were included, as they were collected and analysed independently. They are cited as one study throughout this paper.

We found good agreement between the authors screening the abstracts for eligibility, with a Cohen’s kappa-value of 0.67 (95% CI: 0.57–0.77, *p* = 0.005).

### Study characteristics

All included studies were observational, however, nine studies had prospective and five studies had retrospective data collection strategies; two studies did not report this. The median study duration was 12 months (range 0.5 months–72 months), and the studies included were conducted in 12 different countries (Table 1).

**Table 1:** Basic characteristics for the studies included

Author, year of publication	Study period	Study months	Country of origin	Number of OHCAs analysed	Study design	Data source for registration of recognition	Data source for clinical OHCA
[26]	01-01-2004-31-05-2005	17	Sweden	250	Observational, prospective data collection	Data	Ambulance records/OHCA registry
[7]	1/1-2004 - 1/9-2004	8	The Nether-lands	285	Observational, prospective.	Data	Ambulance records
[23]	1/1-2004 - 31/12-2004	12	Northern Ireland	167	Observational, prospective data collection	Data	Ambulance records/autopsy report
[27]	1/1-2011 - 31/12-2013	36	Switzerland	1,254	Observational, prospective	Emergency call recordings	Ambulance records
[22]	1/1-2003 - 31/3-2003	3	Australia	738	Observational, retrospective	Data	OHCA registry
[19]	1/1-2000 - 30/6-2000	6	US (MO)	370	Observational, retrospective	Data	Ambulance records
[21]	1/1-2007 - 31/12-2007	12	Norway	140	Observational	Emergency call recordings	OHCA registry
[21]	1/5-2010 - 30/4-2011	12	US (VA)	100	Observational	Emergency call recordings	OHCA registry
[29]	1/3-2010 - 31/8-2010	6	Finland	164	Observational, prospective cohort study	Data	Ambulance records
[30]	1/1-1997 - 31/12-2002	72	Finland	373	Observational, retrospective, prospective data collection	Data	OHCA registry
[6]	1/1-2011 - 31/12-2011	12	US (WA)	476	Observational, retrospective cohort study	Emergency call recordings and data	N/A
[14]	1/1-2004 - 01/04-2004	3	Taiwan	199	Observational, retrospective	Data	Ambulance records
[25]	1/1-1996 - 31/12-1996	12	Finland	679	Observational, prospective data collection	N/A	Ambulance records
[15]	1/1-2011 - 31/12-2011	12	Italy	142	Observational, Retrospective cohort study	Data	Ambulance records
[28]	15/5-2012 - 31/5-2012	0.5	France	82	Observational, prospective	Emergency call recordings	Ambulance records
[24]	1/5-2009 - 1/10-2009^a^	17	Canada	1,536	Observational, prospective cohort study	Emergency call recordings	OHCA registry

^a^ = In one of the study centres (Ottawa) the study period was 1/1-2008 - 01/02-2009. US: United States, NO: Norway, OHCA: Out-of-Hospital Cardiac Arrest, N/A: Not available, MO: Missouri, VA: Virginia, WA: Washington

### Recognition

The median sensitivity of OHCA recognition in the included studies was 73.9% (range: 14.1–96.9%) (Table 2). Five studies reported the PPV of OHCA recognition.[14, 19, 22–24] Two studies provided the amount of false positives and true positives, which made it possible to calculate the PPV.[7, 25] The median PPV was 67.4% (range: 58.4–97.9%) (Table 2).

**Table 2:** Main outcome measures, describing the incidence of OHCA in the study population, sensitivity and PPV

Author, year of publication	Incidence^a^(OHCA analysed/100,000/year)	Sensitivity of OHCA recognition	PPV of OHCA recognition
[26]	32.5	20.0%	N/A
[7]	32.9	71.0%	*76.0%
[23]	120.5	68.9%	63.5%
[27]	55.7	71.0%	N/A
[22]	86.8	76.7%	58.4%
[19]	N/A	68.3%	65.0%
[21]	22.5	77.0%	N/A
[21]	49.5	82.0%	N/A
[29]	12.4	82.3%	N/A
[30]	6.1	79.4%	N/A
[6]	35.3	80.0%	N/A
[14]	30.0	96.9%	97.9%
[25]	129.3	*82.9%	*85.3%
[15]	60.0	14.1%	N/A
[28]	30.3	61.0%	N/A
[24]	40.8	65.9%	67.4%

^a^ = Value calculated from information in the study. US: United States, NO: Norway, OHCA: Out-of-Hospital Cardiac Arrest, PPV: Positive predictive value, N/A: Not available

### Definition of “recognised OHCA”

The definition of a “recognised OHCA”, corresponding to the threshold for a positive diagnostic test, differed greatly among studies. Six studies relied exclusively on dispatch codes.[14, 15, 19, 22, 23, 26] Three studies defined the outcome by specific wordings from the emergency call recordings.[24, 27, 28] Two studies used a combination of dispatch codes and emergency calls.[21] One study used a combination of words stated in the emergency call recording, written report and upgrades of emergency responses.[6] Another study used the dispatch of two ambulances as a definition for recognition.[7] Finally three studies did not report the specific definition (Table 3).[25, 29, 30].

**Table 3:** Criteria for out-of-hospital cardiac arrest to be considered recognised

Author, year of publication	Dispatch code	DA-CPR offered	Words stated in emergency call indicating OHCA	Dispatch of two ambulances	Combination of response upgrade and information from the written report indicating OHCA	N/A
[26]	✓					
[7]				✓		
[23]	✓					
[27]		✓	✓			
[22]	✓					
[19]	✓					
[21]	✓	✓	✓			
[21]	✓	✓	✓			
[29]						✓
[30]						✓
[6]		✓	✓		✓	
[14]	✓					
[25]						✓
[15]	✓					
[28]			✓			
[24]		✓	✓			

NO: Norway, US: United States, OHCA: Out-of-Hospital Cardiac Arrest, DA-CPR: Dispatcher Assisted Cardiopulmonary Resuscitation, N/A: Not available

### Study population

The study populations varied in size, with a median of 267.5 OHCA patients (range: 82–1,536). The median incidence of OHCAs analysed/100,000/year in the studies was 35.3 (range: 6.1–129.3) (Table 2). Different exclusion criteria were used for defining each study population. Some populations were defined by patient-related factors such as origin of OHCA or first recorded heart-rhythm, others by call-related factors, such as the patient not being in cardiac arrest at the time of the call or the caller not being at the site of the emergency (Table 4).

**Table 4:** Reported exclusion criteria in the individual studies

Author, year of publication	Exclusion Criteria																
	Age < 18	Trauma/Non cardiac origin	Non-Shockable rhythm	Unwitnessed	No AED use	Caller not at site	DOA	ALS-care not initiated	Inter-hospital transfer	EMS-witnessed	Patient not in OHCA during call	OHCA in HCF	Medical personnel performing CPR	Police, Fire-fighter or GP on duty	EMDC by passed	Secondary emergency call	Interrupted call
[26]										✓	✓						
[7]		✓				✓	✓	✓			✓			✓		✓	
[28]		✓															
[27]	✓	✓								✓							
[22]									✓	✓					✓		
[19]																	
[21]	✓					✓					✓	✓	✓				✓
[21]	✓					✓					✓	✓	✓				✓
[29]			✓	✓						✓							
[30]		✓	✓	✓													
[6]	✓	✓								✓		✓			✓		
[14]	✓	✓															
[25]		✓								✓							
[15]	✓^a^																
[28]	✓^b^				✓	✓					✓						
[14]		✓					✓			✓							

^a^ = Age < 17, ^b^ = Age < 16. US: United States, NO: Norway, ALS: Advanced Life Support, AED: Automated External Defibrillator, OHCA: Out-of-Hospital Cardiac Arrest, EMS: Emergency Medical Services, HCF: Health care facility, CPR: Cardiopulmonary Resuscitation, GP: General Practitioner, VF: Ventricular fibrillation, EMDC: Emergency Medical Dispatch Centre, DOA: Dead on arrival

### EMS system/dispatch centre specific characteristics

Eleven studies reported dispatcher-assisted CPR as being mandatory.[6, 14, 21, 23, 24, 26–30] Five studies reported using the Medical Priority Dispatch System/Advanced Medical Priority Dispatch System (MPDS/AMPDS) as a decision tool [19, 21–23, 26], four used a Criteria Based Dispatch (CBD) decision tool [21, 25, 27], whereas one setting used no decision tool.[15] The remaining seven studies reported use of local protocols of different kinds.[6, 7, 14, 24, 28–30] The dispatchers in the different studies had a variety of professional backgrounds for handling medical emergency calls. Six studies reported that their medical dispatchers were health care personnel (nurses, paramedics, emergency medical technicians), who had further training in dispatching.[7, 14, 15, 19, 21, 27] Two studies had non-health care personnel, with further training in dispatching [22, 24], and the remaining eight studies did not report the professional background.[6, 21, 23, 25, 26, 28–30] The duration of additional training in medical dispatching varied from 32 h to 1.5 years (Table 5).

**Table 5:** Descriptive characteristics for the different EMS systems reporting results for the studies included

Author, year of publication	DA-CPR provided	Decision tool	Medical dispatchers’ educational background	Medical dispatchers’ training
[26]	Yes	MPDS	N/A	N/A
[7]	Yes	Local protocol	Health care personnel	Emergency call/dispatching
[23]	Yes	AMPDS	N/A	N/A
[27]	Yes	CBD	Health care personnel	Annual education (40 h), regular evaluations
[22]	N/A	MPDS	Non-Health care personnel	Emergency call/dispatching
[19]	N/A	AMPDS	Health care personnel	trained in system status management and certified Emergency Medical Dispatcher
[21]	Yes	CBD	Health care personnel	Emergency call/dispatching
[21]	Yes	MPDS	N/A	Certified/qualified medical dispatcher
[29]	Yes	Local protocol	N/A	Emergency call/dispatching (1.5 years)
[30]	Yes	Local protocol	N/A	N/A
[6]	Yes	Local protocol	N/A	Emergency medical training (32 h), annual education, regular evaluations
[14]	Yes	Local protocol	Health care personnel	Emergency call/dispatching (32 h)
[25]	Yes	CBD	N/A	Emergency medical training (228 h), additional training (892 h)
[15]	N/A	None	Health care personnel	N/A
[28]	Yes	Local protocol	N/A	N/A
[24]	Yes	Local protocol	Non-Health care personnel	Unspecified training (6 weeks)

EMS: Emergency medical services, US: United States, NO: Norway, DA-CPR: Dispatcher assisted cardiopulmonary resuscitation, CBD: Criteria Based Dispatch, (A)MPDS: (Advanced) Medical Priority Dispatch, N/A: Not available

### Risk of bias within studies

**Fig. 3:**
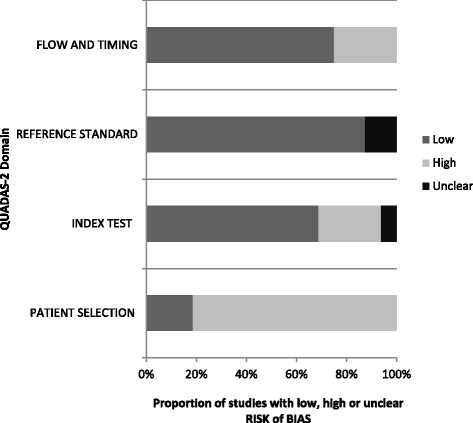
Summary of results from QUADAS-2 risk of bias assessment

The risk of bias within studies was evaluated for all included studies using the QUADAS-2 tool for quality assessment of diagnostic accuracy studies (Fig. 3, Table 6). The main risk of bias in the included studies was present in the “Patient Selection” domain (13 of 16 studies had high risk of bias), as many of the studies had inappropriate exclusions of patients, for the evaluation of OHCA recognition as a diagnostic test (see Table 5). Furthermore, four studies showed high risk of bias in regards to the “Index Test” domain, which was mainly caused by insufficient reporting of threshold for a positive test (the definition of a “recognised OHCA”). In 14 of 16 studies, the risk of bias assessment showed low risk of bias for the “Reference Standard” domain. The clinical diagnosis of OHCA, which was used as the reference standard, was reported in high quality from cardiac arrest registries or ambulance records in the majority of studies.

**Table 6:** Detailed results from QUADAS-2 risk of bias assessment

Author, year of publication	RISK OF BIAS			
	PATIENT SELECTION	INDEX TEST	REFERENCE STANDARD	FLOW AND TIMING
[26]	✓	✓	✓	✘
[7]	✘	✓	✓	✓
[23]	✘	✓	✓	✘
[27]	✘	✓	✓	✘
[22]	✓	✘	✓	✓
[19]	✓	✓	✓	✓
[21]	✘	✓	✓	✓
[21]	✘	✓	✓	✓
[29]	✘	✘	✓	✓
[30]	✘	✘	✓	✓
[6]	✘	✓	?	✘
[14]	✘	?	?	✓
[25]	✘	✘	✓	✓
[15]	✘	✓	✓	✓
[28]	✘	✓	✓	✓
[24]	✘	✓	✓	✓

✓ = Low Risk ✘ = High Risk ? = Unclear Risk

NO: Norway, US: United States of America

## Discussion

This systematic review, describing recognition of OHCA during emergency calls, included 16 observational studies with 6,955 patients in total. Our main findings were a median sensitivity of OHCA recognition of 73.9%. Sensitivity and PPV of OHCA recognition, as well as the incidence of OHCA in the studied populations, varied greatly between the included studies. There were great heterogeneity among studies – especially in the selection of study population and the definition of “recognised OHCA”.

A median sensitivity of 73.9% for recognition of OHCA is high compared to other time-critical conditions such as stroke where recognition rates from 31–66% are reported. [31–33] To obtain a sensitivity of 100%, EMS systems would risk increasing the amount of cases where ambulances are dispatched to patients not in cardiac arrest, also referred to as over-dispatching. The range in sensitivity (14.1–96.9%), in combination with the varying definitions of study population and “recognised OHCA”, questions the comparability of results among the studies included. The variation in PPV, between the studies where it was reported, was also substantial. The PPV is an important performance measure to assess in relation to the sensitivity as it illustrates the amount of over-dispatching in a system. However, a degree of over-dispatching must be accepted for OHCA patients.

The organisation of the EMS may affect the performance of the medical dispatchers. Different dispatch tools are used to assist the medical dispatchers. The most common are the AMPDS/MPDS and the CBD. One study compared the two systems and found no difference in recognition of OHCA.[21] The professional background and training of the medical dispatchers varied greatly. This could potentially affect performance, but no recommendations for professional background or amount of additional training for medical dispatchers exists in international guidelines.[34, 35].

### Limitations at study and outcome level

The definition of a “recognised OHCA” is essential in studies reporting OHCA recognition during emergency calls. It corresponds to the threshold for a positive test in a diagnostic test accuracy study. The definitions in the included studies often depended on the data source for assessment of recognition, with specific dispatch codes or response types in studies using EMS data, and specific wordings or the provision of dispatcher-assisted CPR in studies evaluating emergency calls. Such definitions cause low risk of bias in individual studies, but impair the comparability across studies. Three studies did not report the specific criteria for a “recognised OHCA”.[25, 29, 30] This hinders the interpretation of results and is discouraged. The validity of dispatch codes as a data source is dependent on the specific design of the EMS system and the dispatch tool, from which the dispatch codes arise, as well as the background and training of the personnel performing the registration. Emergency calls can provide high-quality data, but the validity of emergency calls, as data source is highly dependent on the method for collecting such data. There is a large risk of confirmation bias when retrospectively evaluating the emergency calls, especially regarding the objectivity of the investigator, and whether specific wordings were rigorously pre-specified. The evaluation of emergency calls complies with the risk that dispatch codes are not registered during the very hectic emergency call process. Dispatch codes can provide large amounts of data, compared to emergency call recordings, which is a very time-consuming way of collecting data. Furthermore, the use of dispatch codes as data source makes it possible to collect the amount of false positive cases and report the PPV. One way of obtaining a proxy for PPV when using emergency calls as data source is to evaluate a random sample of calls not classified as OHCA by the dispatcher, and then extrapolate the results to correspond to the total amount of calls, as presented in another study.[7].

The selection of population for a study on OHCA recognition is essential for the generalizability of the results. In the studies included in this review, the predefined exclusion criteria varied greatly, which is expressed clearly in the very different incidence rates across studies ranging from 6.1 to 129.3 OHCAs analysed/100,000/year. It was common among the studies to exclude EMS-witnessed cases, which seems obvious as these were not in cardiac arrest during the emergency call. Some studies also excluded cases where recognition was “not detectable”, for example cases where the caller was not at the site of the patient or the call was interrupted.[7, 21, 28] Such a selection seems relevant, but it includes an extent of subjectivity in evaluating which OHCAs that were “not detectable”, which could affect the final results. Certain studies reported recognition among a very specific study population, which was limited by specific heart rhythm or the use of AEDs.[28–30] Such populations are very narrow, leading to low incidence rates. No studies excluded all cases where bystander CPR was initiated prior to the call. Not excluding all of these cases may lead to selection bias and result in falsely high OHCA recognition, since these cases would be categorised as recognised, even though the dispatcher did not participate in the recognition.

### Limitations at review level

Despite the strict methodology implied by reporting according to the PRISMA guidelines, our study contains some limitations. The main limitation is the lack of meta-analysis in this study. However, a meta-analysis would be uninformative due to the heterogeneity of the included studies. The included studies reported OHCA recognition from a variety of different dispatch centres, with variations in decision tools as well as professional background and level of training of the personnel (see Table 5). Furthermore, the included studies had very different criteria for the categorisation of a “recognised OHCA” (see Table 3) and dissimilar study populations (see Table 4). When considering OHCA recognition as a diagnostic test, this would correspond to heterogeneity in the specific tests that were evaluated, the threshold for positive test, and the study population. To include such heterogeneous studies in a meta-analysis would be uninformative and is discouraged by the Cochrane Collaboration.[36] The International Liaison Committee on Resuscitation reached the same conclusion in the Consensus on Science and Treatment Recommendations for recognition of OHCA.[37].

Secondly, our study is limited by the exclusion of interventional studies with OHCA recognition as outcome variable. These are excluded because they study the effect of specific interventions on OHCA recognition. Such studies are very important for future improvements in emergency medical dispatch, but not the objective of this systematic review.

Finally, as the OHCA recognition rate is an indicator of performance for EMS systems, a substantial degree of reporting bias must be expected for two reasons: 1) poorly performing systems may be reluctant to publish their results, and 2) the better performing systems may be the only systems with resources to measure this and therefore data to publish.

### Future aspects

A recognition rate of 75% within one minute and dispatcher-assisted CPR rate of 75%, in cases where the dispatcher has the opportunity of assessing consciousness and breathing, has been suggested as performance standard following the 2015 Utstein meeting on *“implementation of best practices in community resuscitation”*.[38] Recognition of OHCA during the emergency call is a modifiable factor from an EMS organisational aspect.[7] In order to recognise an OHCA the medical dispatcher must have the right competences and the relevant tools for support.[35, 39] Studies have shown significant improvements in OHCA recognition and dispatcher-assisted CPR due to different interventions.[40–42].

**Fig. 4:**
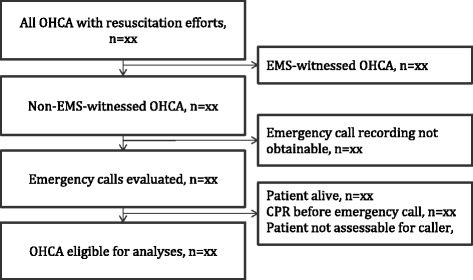
Suggested flowchart for future reporting of out-of-hospital cardiac arrest recognition. EMS: Emergency medical services, OHCA: Out-of-hospital cardiac arrest, CPR: Cardiopulmonary resuscitation

In order to use OHCA recognition as a benchmark to compare the results of interventions and improve EMS systems, it is necessary that the reporting of results is uniform and thus comparable. Uniform reporting of data from OHCA has been established and improved in the Utstein style guidelines since 1991.[43, 44] However, guidelines for uniform reporting on variables concerning emergency medical services and medical dispatch, has not yet been included, despite several efforts.[6, 27, 45] Such a guideline should specify the definition of “recognised OHCA” in order to make results interpretable for the readers and comparable between organisations. Furthermore, it should standardise the appropriate study population for assessment of OHCA recognition. We believe that this population should consist of the cases where the recognition of OHCA will have the most relevant clinical implication. To uniform the reporting of recognition, we suggest the following:

- Recognition should be assessed by evaluating emergency call recordings
- Recognised OHCAs should be defined as cases where the caller or the dispatcher, expressed the presence of "OHCA", or the need for "CPR" or an "AED"
- The following cases should be excluded: EMS-witnessed, missing/corrupted emergency call recording, cases where the patient was obviously alive during the call, cases where bystander CPR was initiated prior to the emergency call, and cases where the caller was unable to assess the patient
- The data collection should be reported in a standardized flowchart (Fig. 4) and results should include incidence, sensitivity, and PPV if possible

## Conclusion

In conclusion, this systematic review of observational studies, report a median sensitivity for OHCA recognition across studies of 73.9%. Great heterogeneity in the definitions of study population and “recognised OHCA”, lead to insufficient comparability of the results reported in the included studies. Recognition of OHCA is an important and modifiable factor in the chain of survival, and it should serve as a performance measure for EMS systems. However, uniform reporting and transparency is needed.

## Abbreviations

(A)MPDS: (Advanced) medical priority dispatch system
AED: Automated external defibrillator
CBD: Criteria based dispatch
CPR: Cardiopulmonary resuscitation
OHCA: Out-of-hospital cardiac arrest
PPV: Positive predictive value
